# Oropouche Virus: Clinical, Epidemiological, and Molecular Aspects of a Neglected Orthobunyavirus

**DOI:** 10.4269/ajtmh.16-0672

**Published:** 2017-05-03

**Authors:** Jorge Fernando Travassos da Rosa, William Marciel de Souza, Francisco de Paula Pinheiro, Mário Luiz Figueiredo, Jedson Ferreira Cardoso, Gustavo Olszanski Acrani, Márcio Roberto Teixeira Nunes

**Affiliations:** 1 Evandro Chagas Institute, Ministry of Health, Pará, Brazil; 2 Virology Research Center, School of Medicine of Ribeirao Preto of University of São Paulo, São Paulo, Brazil; 3 MRC-University of Glasgow Centre for Virus Research, Glasgow, Scotland; 4 Faculty of Pharmaceutical Sciences of Ribeirão Preto, University of São Paulo, São Paulo, Brazil; 5 Universidade Federal da Fronteira Sul, Passo Fundo, Rio Grande do Sul, Brazil; 6 Department of Pathology, Center for Biodefense and Emerging Infectious Diseases, University of Texas Medical Branch, Galveston, Texas

## Abstract

Oropouche virus (OROV) is an important cause of arboviral illness in Latin American countries, more specifically in the Amazon region of Brazil, Venezuela and Peru, as well as in other countries such as Panama. In the past decades, the clinical, epidemiological, pathological, and molecular aspects of OROV have been published and provide the basis for a better understanding of this important human pathogen. Here, we describe the milestones in a comprehensive review of OROV epidemiology, pathogenesis, and molecular biology, including a description of the first isolation of the virus, the outbreaks during the past six decades, clinical aspects of OROV infection, diagnostic methods, genome and genetic traits, evolution, and viral dispersal.

## Introduction

Oropouche virus (OROV) is one of the most common arboviruses that infect humans in Brazil. It is estimated that since the first isolation of the virus in 1955, it has affected more than half a million people. However, the exact number of cases is difficult to determine, because the infection is underreported due to the similarity of symptoms with other arboviral febrile illnesses, such as Dengue, Zika, Chikungunya, and Mayaro fevers. The lack of an exact diagnosis in hospitals and health-care centers hinders the proper epidemiological notification, which is the principal reason why the estimated number of cases is lower than what is probably occurring in the population, especially in the Amazon-endemic region.

OROV is the causative agent of Oropouche fever, a febrile arboviral illness that is frequently associated with the Brazilian–Amazon region.^1^ The first case of OROV disease was described in Trinidad and Tobago in 1955: the virus was isolated from the blood of a febrile forest worker (strain TRVL 9760), a resident of a village called Vega de Oropouche, 3 miles north of Sangre Grande.^2^ Another strain of OROV was isolated from a pool of 177 *Coquillettidia venezuelensis* (Theobald) mosquitoes collected in Bush Bush Forest, Nariva Swamp, Trinidad, on October 18, 1960. This second strain (TRVL 35111) was antigenically closely related to the prototype strain TRVL 9760 by complement fixation (CF) and neutralization (NT) tests.^2^ The virus was first isolated in Brazil in 1960 from the blood of a sloth, *Bradypus trydactilus*, captured in a forested area during the construction of the Belém-Brasilia highway and also from a pool of *Ochlerotatus serratus* mosquitoes caught near the same area.^3^

In the following year, the virus was again detected in Belém City, the capital of Pará State, northern Brazil. On that occasion, a large epidemic of Oropouche fever was reported in Belém with an estimated 11,000 people affected.^4^ With that outbreak, OROV demonstrated its epidemic potential and many other outbreaks have been described subsequently in urban areas in the states of Acre, Amapá, Amazonas, Maranhão, Pará, Rondônia, and Tocantins, as well as other South American countries, such as Panama in 1989 and at the Amazon region of Peru between 1992 and 1994.^1^,^5^,^6^

More recently, OROV was reported in the municipalities of Parauapebas, Porto de Moz, Igarapé Açu, Magalhães Barata, and Maracanã, in Pará State, northern Brazil; the last three were located in the Bragantina area, the region where the virus was first detected in 1970.^7^,^8^ In 2009, the last OROV outbreak was reported in the municipalities of Altamira and Santa Barbara, Pará State, and subsequently in Mazagão in the state of Amapá, Brazil.^9^

**Figure 1.**
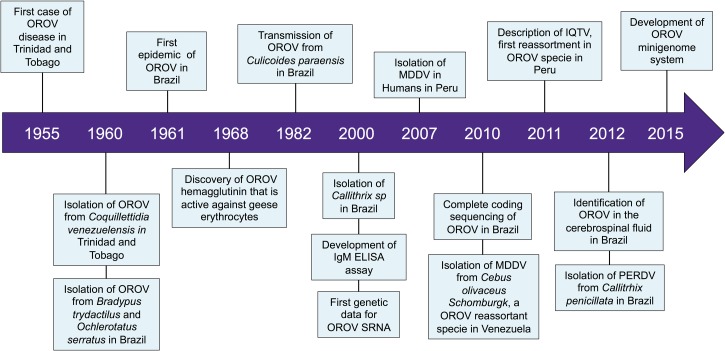
Time line of advances in Oropouche virus research.

In this report, we review the past 60 years of OROV research, providing a brief overview of some of the key milestones that have been achieved since the original isolation of the virus (Figure 1

). We also discuss some of the main current research directions and key goals for the future, with an emphasis on taxonomy, epidemiology, transmission cycles, evolution, and progress on the understanding of the pathogenesis.

## Taxonomy and Classification

OROV is a member of the family *Bunyaviridae*, genus *Orthobunyavirus*, the largest genus of RNA viruses with over 170 named viruses corresponding to 18 different serogroups and 48 species complexes.^10^,^11^ The OROV classification was originally made using serological methods, such as CF, hemagglutination inhibition (HI), or neutralizing (NT) tests.^12^ These methods have been used for grouping viruses by their antigenic relationship. OROV belongs to the Simbu serogroup, which includes 22 officially recognized viruses that have been grouped into seven different species complexes: Akabane, Manzanilla, Oropouche, Sathuperi, Simbu, Shamonda, and Shuni,^11^ as well as several other recently described viruses that have not yet been assigned to a species or serocomplex (Table 1). With the advent of molecular methods and next generation sequencing, full genomic sequences have been determined, improving the taxonomy of viruses, including members of the genus *Orthobunyavirus*.^13^ Currently, the Simbu serogroup is composed by two phylogenetic subclades: subclade A, which includes Oropouche and Manzanilla orthobunyaviruses, and subclade B, constituted by Simbu, Shuni, Shamonda, Sathuperi, and Akabane viruses.^14^ Hemagglutinin antigens can be prepared to OROV from brain and serum samples of infected hamsters, which have been used in epidemiological surveillance for precise serological diagnostic of OROV infections since 1985.^15^,^16^

OROV replicates in numerous cell cultures, including C6/36, Vero, BHK-21, MA III, LCM-MK2, and primary chicken embryo fibroblasts, causing a cytopathic effect from subtotal to total destruction of the cell monolayer, depending on the multiplicity of infection used and time postinfection.^16^ OROV is sensitive to sodium deoxycholate, which reduces the ability of the virus to infect the host cells by destroying the envelope glycoprotein, a viral structure that is directly associated with virus–host interaction.^14^,^17^,^18^

## Viral Structure, Genome, and Replicative Cycle

Although no specific ultrastructural studies of OROV in human tissues have been published to date, it is probable that this viral agent exhibits particles with similar morphological characteristics to other members of the *Orthobunyavirus* genus. Ultrastructural studies of La Crosse virus have shown that virus particles are spherical, measuring between 80 and 110 nm in diameter, surrounded by a lipid envelope.^19^ Internally, the viral particle contains three single-stranded negative sense segments of genomic RNA of different sizes that are individually connected to the L protein (viral RNA-dependent RNA polymerase) and which are surrounded by the nucleocapsid (N) protein, forming three ribonucleoproteins.^20^

**Figure 2.**
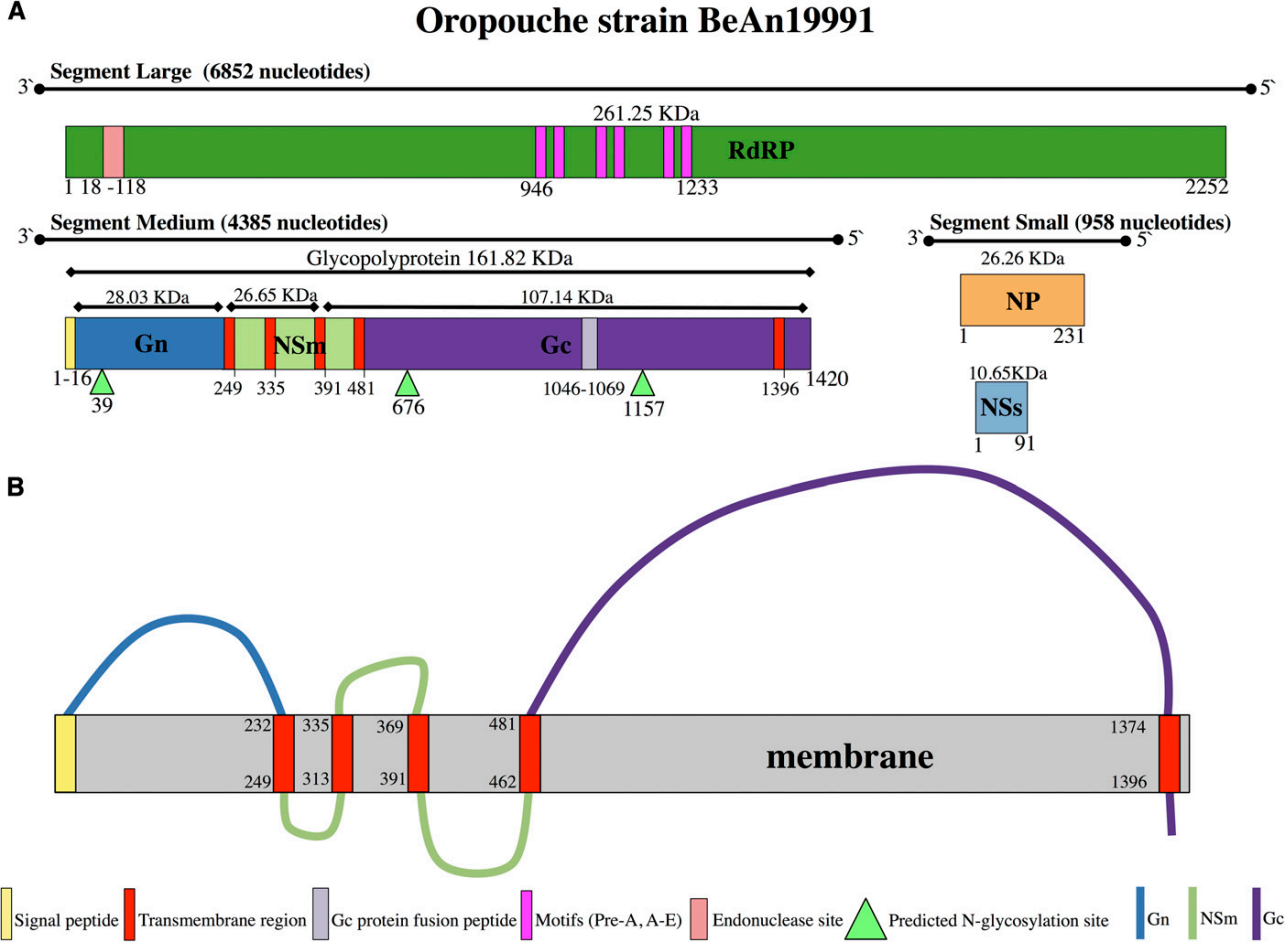
Schematic representation of (**A**) genomic organization and (**B**) topology of glycoprotein of Oropouche virus strain BeAn 19991.

The genomic segments are named small (SRNA), medium (MRNA), and large (LRNA), according to their respective molecular sizes. The partial viral genome for the Brazilian prototype OROV strain BeAN 19991 was sequenced, and recently the complete genomic sequences for the three segments, including the full noncoding regions (NCRs) was described, showing a SRNA segment of 958 nucleotides, a MRNA with 4,385 nucleotides and a LRNA with 6,852 nucleotides in length (Figure 2

).^21^–^23^ The coding sequences of the three genomic segments are flanked by two terminal NCRs namely 5′ and 3′ NCRs, that have different number of nucleotides in length, but have eleven nucleotides that are highly conserved between the three RNA segments. These regions are complementary to each other in a typical arrangement that provides a circularization in the genomic RNA that is essential for the activity of this region as replication and transcription promoters, as indicated recently in a minigenome system assay that introduced mutations in the NCRs.^22^

The LRNA contains one open reading frame (ORF) that encodes the L protein, an RNA-dependent RNA polymerase. The L protein has a molecular weight of 261.25 kDa and is associated to the three viral RNA segments.^24^ The MRNA contains a single ORF that encodes a large polyprotein that is cleaved after or during translation, yielding three viral proteins, two structural surface glycoproteins: Gn (28.03 kDa) and Gc (107.14 kDa) and a nonstructural protein named NSm (26.65 kDa).^11^ The SRNA segment encodes a structural nucleocapsid protein (26.26 kDa) and a nonstructural NSs protein (10.65 kDa), in two overlapping ORFs. Recently, the rescue of recombinant OROV viruses by reverse genetics lacking these nonstructural proteins demonstrated that NSm is dispensable for virus replication in mammalian and mosquito cells, whereas NSs is an important virulence gene, acting as a type I interferon (IFN) antagonist.^25^

The details of the OROV replication cycle are still unknown. Progeny production is observed 10 hours postinoculation of the virus in HeLa cells, with a peak after 24 hours.^26^ The interaction between the virus particle and the cell receptor is most likely mediated by the surface glycoproteins Gn and Gc, and the virus enters HeLa cells by endocytosis mediated by clathrin-coated vesicles, whereas the release of viral particles from the endosome is dependent on endosomal acidification.^26^

One important cytopathic effect observed after OROV replication in HeLa cells is the induction of apoptosis, which was detected at 36 hours postinfection.^26^ Release of cytochrome c and activation of caspases 9 and 3 were detected, and apoptosis occurs without affecting the viral load, indicating that it might be important during the replication cycle of OROV. The same work suggested that viral protein synthesis is necessary for the induction of apoptosis, indicating that one or more viral proteins might be involved in this mechanism.

## Geographical Distribution of OROV

Thus far, the only reported cases of Oropouche (ORO) fever have occurred in Brazil, Panama, Peru, and Trinidad and Tobago. In Brazil, since the first isolation of the virus in 1955 until 1980, OROV caused several epidemics apparently restricted to the State of Pará, northern Brazil, reaching different municipalities of distinct mesoregions: metropolitan area of Belém (municipalities of Belém, Ananindeua, Benfica, Caraparu, Castanhal, and Santa Isabel); northeast (Abaetetuba, Augusto Correia, Baião, Bragança, Capanema, Curuçá, Tomé-Açu, Vigia, and Viseu); southeast (Itupiranga); lower Amazon (Belterra, Mojuí dos Campos, and Santarém); and Marajó Island (Porto de Moz). During this period, only the southwest mesoregion did not report cases or ORO fever.^4^,^27^–^30^

Between 1981 and 1996, outbreaks of ORO fever were registered in the state of Pará (Oriximiná, mesoregion low Amazon; Brazil Novo and Altamira, mesoregion southeast); as well as in other states, such as in the cities of Manaus, Novo Airão, and Barcelos (Amazonas State), Mazagão (Amapá State), Xapuri (Acre State), Ariquemes, Machadinho, and Ouro Preto d'Oeste (Rondônia State), Porto Franco and Estreito (Maranhão State), and Tocantinópolis (Tocantins State).^1^,^15^,^16^ In 2000 and 2010, the virus was isolated from sylvatic monkeys (*Callithrix penicillata*) on two different occasions in Minas Gerais State, southeast Brazil.^22^,^31^

In 2003 and 2004, outbreaks of ORO fever were detected in the municipalities of Parauapebas, Pará State (mesoregion east) and Porto de Moz (middle region of the lower Amazon), respectively.^7^ In 2006, additional OROV epidemics were registered in Pará State, in the cities of Maracanã, Igarapé-Açu, Magalhães Barata, and Viseu located in Bragantina area of northeast Pará, demonstrating the reemergence of the virus after 26 years of epidemiological silence in the region.^8^ In 2009, OROV reemerged in Pará State, more specifically in the municipalities of Santa Bárbara (metropolitan region of Belém), Altamira (mesoregion southeast), and in Mazagão, state of Amapá.^9^

Outside of Brazil, epidemics have been reported in Panama and Peru. The outbreak in Panama was recorded in 1989 in the Village of Bejuco, located approximately 50 km west of Panama City, capital of the country. In Peru, ORO fever was documented in 1992, when the virus caused an outbreak in Iquitos City.^5^,^6^ Furthermore, two other outbreaks of OROV were recorded in the Peruvian Amazon in 1994, in the cities of Puerto Maldonado and Madre de Dios.^32^

## Epidemic Dispersion and Seasonal Distribution

ORO fever cases have been reported in different locations in a large geographical area, including both in South and Central Americas. During outbreaks, a representative epidemic dispersion process is observed, with transmission to several locations situated close to the region where the virus was first detected. This phenomenon was observed in outbreaks that occurred in several municipalities in the states of Pará and Rondônia, Brazil.^7^,^8^ The dispersal pattern of the virus is probably related to the movement of infected viremic people to locations where the potential urban vector *Culicoides paraensis* can be found.^3^

ORO fever occurs predominantly in the rainy season, due to the increase in the breeding sites of the vector population. In Brazil, more specifically in the Brazilian Amazon region, the dry season corresponds to the months from July to December, whereas the rainy season occurs between January and June.^3^ Although less often, some OROV epidemics have been reported during the dry season, probably due to the high population density of *Culicoides paraensis* during previous rainy period. Furthermore, the occurrence of outbreaks is also related to the increase of susceptible human population not previously exposed to the virus. In general, decreasing of outbreaks is associated with the beginning of the dry season and the decreasing of midge density.^16^

## Transmission Cycles

**Figure 3.**
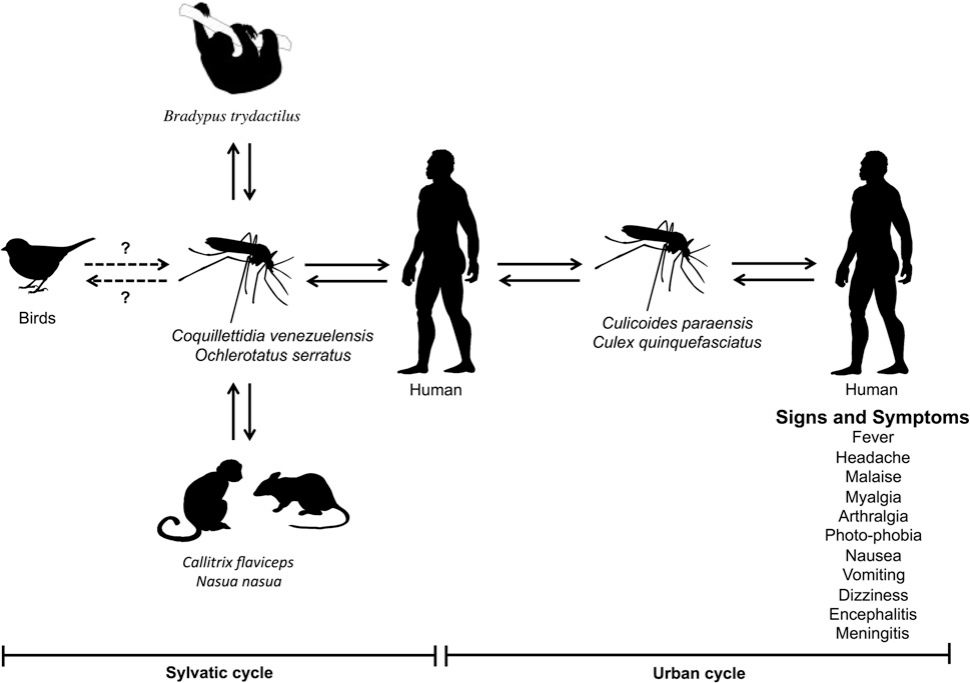
Transmission cycles of Oropouche virus.

Studies conducted by the Evandro Chagas Institute suggest that OROV is maintained in nature by two distinct cycles: sylvatic and urban (Figure 3

).^33^ In the sylvatic cycle, there is evidence that pale-throated sloths (*Bradypus tridactylus*), nonhuman primates and some wild birds play a role as vertebrate hosts.^16^,^22^,^31^

The vector of OROV in the forest is still unclear. On two occasions, the agent has been isolated from sylvatic mosquitoes, both in 1960. One isolation was from *O. serratus* collected in the Amazon region of Brazil, and the second was from *Coquilletidia venezuelensis* in Trinidad.^3^,^16^

In the urban or epidemic cycle, humans apparently are the only vertebrates involved, since studies with domestic animals (e.g., dogs, cats, and chickens) excluded the role of these animals in the maintenance of the urban cycle.^16^ Humans are probably also the link between the sylvatic and urban cycles, when humans invade the forest, become infected, and return to urban areas during the viremic period.^16^ Two vectors are commonly found in urban epidemics: the biting midge *Culicoides paraensis* (*Ceratopogonidae*), regionally named as “*maruim*,” and the mosquito *Culex p*. *quinquefasciatus* (*Culicidae*). In laboratory experiments with the *Culex p. quinquefasciatus*, the insect proved to be an inefficient vector of OROV.^34^ Furthermore, Cardoso and others detected the SRNA of OROV in patients and in *Culex quinquefasciatus*, reinforcing the probable participation of *Culex* mosquitoes in the urban cycle of OROV.^35^

The involvement of the biting midge as a vector of OROV is based on experimental studies carried out by Pinheiro and others.^16^ The authors demonstrated the ability of *Culicoides paraensis* to transmit the virus to hamsters after five or more days feeding on blood of viremic patients. These midges have diurnal activity, especially during the twilight period, and are aggressive bitters that are attracted by humans. *Culicoides paraensis*, small insect, is widely distributed in tropical and subtropical areas of the Americas, and is usually found in high density during epidemic periods. The larvae feed on decomposing organic materials, such as trunks of banana trees (genus *Musa*), fruits husks of coconut (*Cocos nucifera*), and Cupuaçu (*Theobroma grandiflorum*) trees, as well as in accumulated debris of trees.^36^

However, the low isolation rate of the virus from *Culicoides paraensis* during epidemics is an intriguing problem. Further studies are necessary to determine if this phenomenon is due to the low susceptibility of the insect to OROV, or if only a small fraction of *Culicoides paraensis* population has the ability to transmit the virus.^37^

## Incubation and Transmission Periods

There is no precise information about the incubation period of ORO fever; however, some observations made during major epidemics suggest that it may vary from 4 to 8 days. In 1981, Pinheiro and others described two laboratory technicians who were accidentally infected with OROV, and showed ORO fever symptoms 3–4 days after infection.^16^ Although the report does not mention the conditions in which the virus was being manipulated, the transmission is thought to have occurred through the respiratory route.^16^

The patient's blood in the acute phase of the illness is infective to the *Culicoides paraensis* for the first 3–4 days from the onset of symptoms, when viremia is high enough to infect biting midges. Experimental studies in hamsters (*Mesocricetus auratus*) demonstrated that the extrinsic incubation period is five or more days.^38^,^39^ No evidence of direct transmission of OROV from one person to another has been reported.^33^

## Incidence

In general, the estimated incidence rates for OROV infection have been determined by seroepidemiological surveys, where groups of families were randomly selected. In this case, a clinical–epidemiological survey was applied to members of each family, and blood samples were collected for virus isolation (acute phase) in newborn mice (2–3 days old) and Vero cells, as well as for the detection of NT, CF, HI, and IgM antibodies (convalescent phase).^3^,^30^

Although the incidence values have not been determined in some outbreaks, a relevant feature of ORO fever is related to a large number of OROV infections reported in all epidemic episodes described so far. Based on official records, the overall cases of ORO fever were estimated at 380,000 cases during the period from 1961 to 2007. It is important to note that in several outbreaks the uniformity in the numerical distribution of cases, as well as in the estimated incidence of cases was not homogeneous. Indeed, the average incidence rate was estimated as 30%. In one of the described epidemics, the proportion of infected individuals showing clinical symptoms of OROV infection reached 63%.^28^

Regarding gender, rates of OROV infections are quite varied. In 1979, during the outbreak in the Bragantina area, northeastern Pará State, females were the most infected group. On the other hand, in the same year, another outbreak occurred in Belém City, where males were the most affected.^16^ In an outbreak reported in Santarém City, Pará State, northern Brazil, the proportion of infected women was twice as high as the male population. In addition, ORO fever affects all age groups, although in some outbreaks, the incidence was greater in children and young adults.^3^,^27^

## Molecular Epidemiology

The first genetic study carried out with OROV was described by Saeed and coworkers in 2000 and introduced the basis for understanding the molecular epidemiology of this viral agent. It suggested the existence of at least three distinct circulating genetic lineages in the Americas: genotypes I, II, and III.^40^ Based on findings from the nucleotide sequences obtained from the *N* gene of 28 OROV strains isolated from different hosts, geographical locations, and different periods of time, it was reported that genotype I was only detected in Trinidad and Tobago, whereas genotype II was restricted to Peru, and genotype III was found in Panama only. In Brazil, only genotypes I and II were detected: genotype I was more often found in eastern Amazon, whereas genotype II in western Amazon region.^40^

In 2000, a strain of OROV was isolated from a new vertebrate host (*Callithrix* sp.) in the municipality of Arinos, Minas Gerais State, southeastern Brazil, and this isolate was classified as genotype III, previously found only in Panama. This finding reinforced the hypothesis that OROV has encountered favorable ecological conditions to allow its spread to other regions outside the original epidemiological area in the Amazon region. Furthermore, this also opened the possibilities for the virus to spread into highly susceptible populations that were residing in these urban areas, increasing the risk of outbreaks.^31^,^41^

Molecular analysis of additional strains isolated during epidemics that occurred between 2003 and 2006 in the municipalities of Parauapebas, Porto de Moz, Igarapé Açu, Maracanã, Magalhães Barata, and Viseu in the Bragantina region (Pará State) demonstrated the co-circulation of genotypes I and II and their association with ORO fever cases.^7^,^8^ However, these results have to be treated with caution as the authors used only partial genetic information from each gene and not complete sequences.

**Figure 4.**
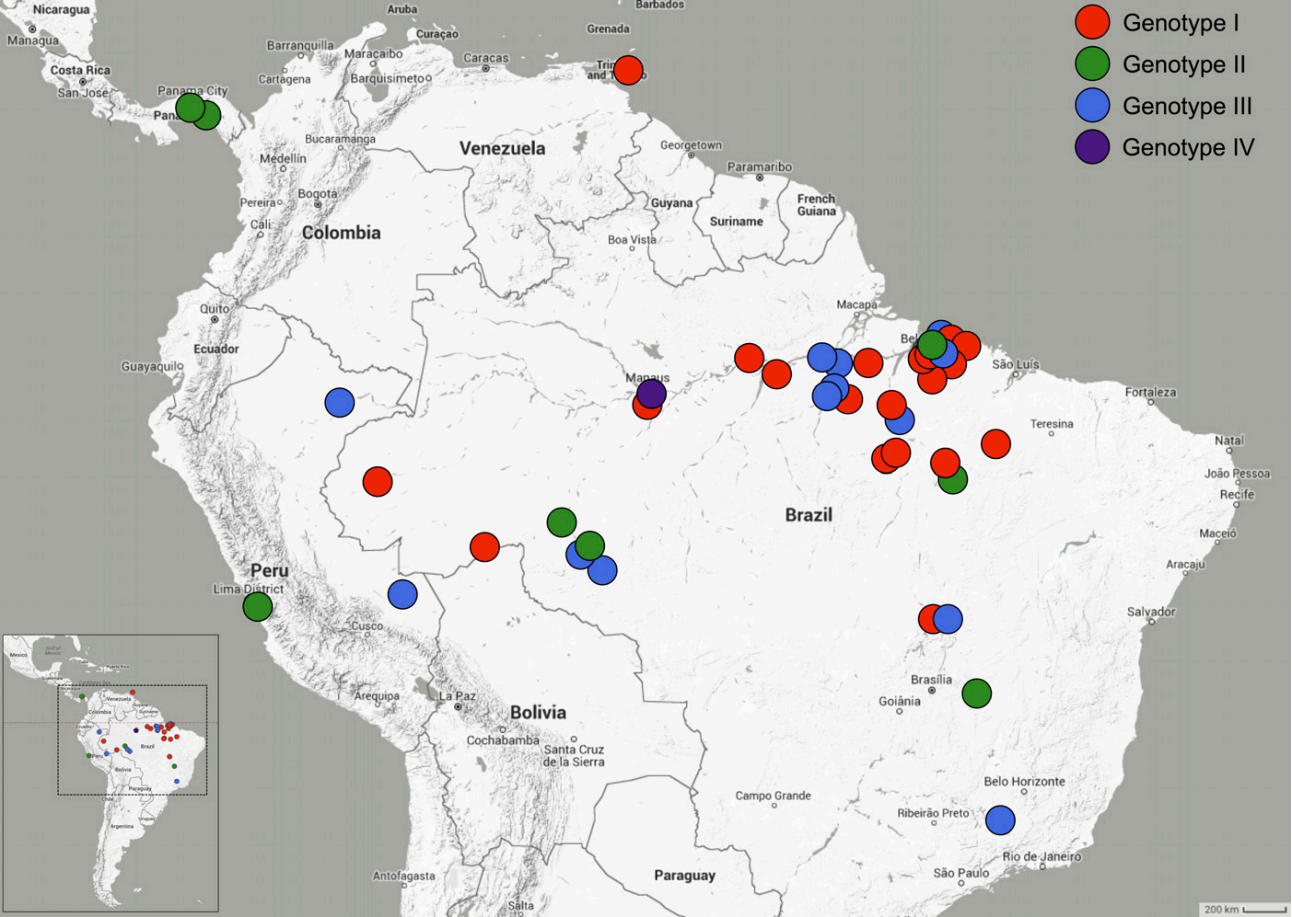
Distribution of genotypes of Oropouche virus in Latin America based on 114 sequences of *N* gene.

In the present study, the appearance of a fourth genotype, based on information obtained from genetic data for the SRNA segment was observed. This new phylogenetic group, isolated from humans during an outbreak in Manaus in the 1980s, was called genotype IV.^9^ Therefore, based on 111 complete coding sequences of the SRNA, it was possible to subdivide OROV into four genotypes and eight subgenotypes. Currently, the geographical distribution of OROV is described as follows: genotype I in Trinidad and Tobago and Brazil; genotype II in Panama, Peru, and Brazil; genotype III detected in Brazil and Peru, and the new genotype IV isolated only in Brazil (Figure 4

).

## Time-Scaled Analysis and Phylogeography of OROV Genotypes

**Figure 5.**
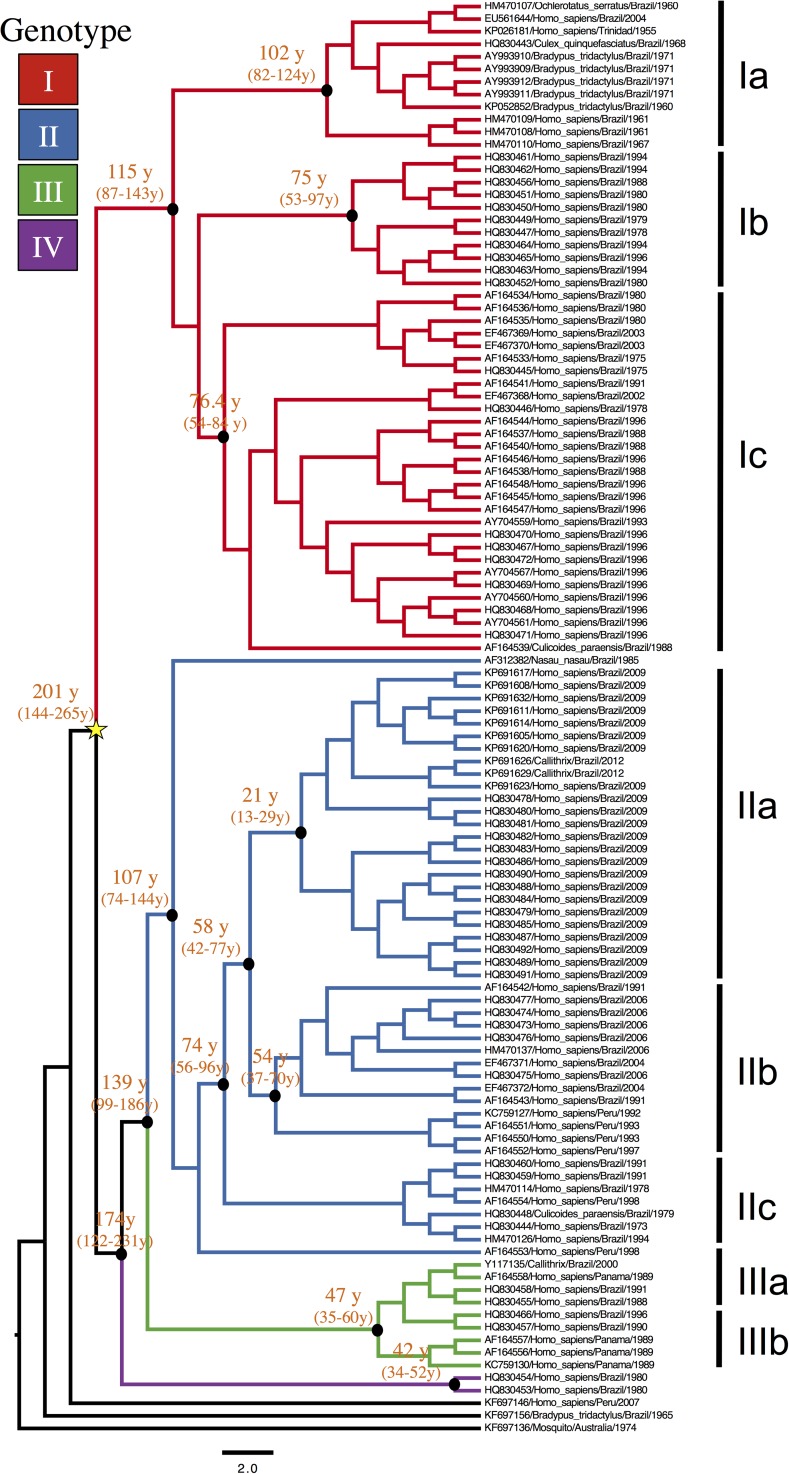
Phylogenetic tree based on the complete nucleotide sequence of the *N* gene of 114 Oropouche virus strains isolated from different hosts, locations, and periods. The main phylogenetic groups are represented by genotypes in color and subgenotypes in left bars. The values above the main nodes represent the dates of emergence of common ancestors, expressed in years before 2012. The arrows indicate the probable date of emergence of genotypes I, II, III, and IV. Numbers in parentheses are value for 95% highest probability density.

Chronological analyses were conducted to investigate the emergence period of OROV in the Americas.^9^ We also determined the nucleotide substitution rate, based on 96 OROV *N* gene sequences (3.7 × 10^−4^ substitutions per site per year), and we used this information to calculate the divergence dates among the strains. The most recent common ancestor for OROV in the Americas was estimated to have emerged around 223 years ago (95% highest probability density [HPD]: 148–342 years) generating the four different genotypes (I, II, III, and IV) that are currently described. Detailed analyses suggest that genotype I was the ancient genotype emerging ∼112 years ago (95% HPD: 95–189 years), whereas genotype II subsequently emerged ∼91 years ago (95% HPD: 59–144 years) and was probably originated from strains isolated in the states of Pará and Rondônia, as well as from strains recently isolated in the Amapá State, in 2009. Genotype IV emerged in the Amazonas State ∼43 years ago (95% HPD: 31–56 years) and genotype III was considered the latest lineage to emerge in Brazil, about 37 years ago (95% HPD: 33–70 years) probably in the Rondonia State 33 years ago (95% HPD: 29–58 years), and in other states from Amazon region (e.g., Acre and Pará States), and emerging almost simultaneously in Panama about 32 years ago (95% HPD: 22–45 years) and, more recently, in Minas Gerais State, out of the official endemic area of OROV transmission (Figure 5

).

Based on results obtained for the *N* gene data by time-scaled analysis (evolutionary rate and emergence date) and epidemiologic data association (date and geographic location of isolation), the possible dispersal event could be predicted for the distinct OROV genotypes in the Americas. Genotype I (originally isolated in Brazil in the municipality of Ipixuna, Pará State, BR 010 Highway, km 94), possibly spread continuously toward distinct regions: initially to several municipalities in western Pará State, and almost simultaneously in Trinidad and Tobago. Later, genotype I moved toward the states of Amazonas and Acre and finally to the eastern Amazon region including Pará, Maranhão, and Tocantins States. Genotype II apparently emerged simultaneously in the states of Amapá, Pará, and Rondônia, as well as in Peru, and dispersed in these places, emerging in the municipality of Mazagão, Amapá State, in 2009. Genotype III emerged in Rondônia State, moving toward Panama and the states of Acre and Maranhão in Brazil, and lately to Minas Gerais State. Genotype IV, apparently more ancient than genotype III, emerged in the city of Manaus, Amazonas State, and apparently is still restricted to that area.

## Genomic Reassortment: Mechanism of Virus Evolution

**Figure 6.**
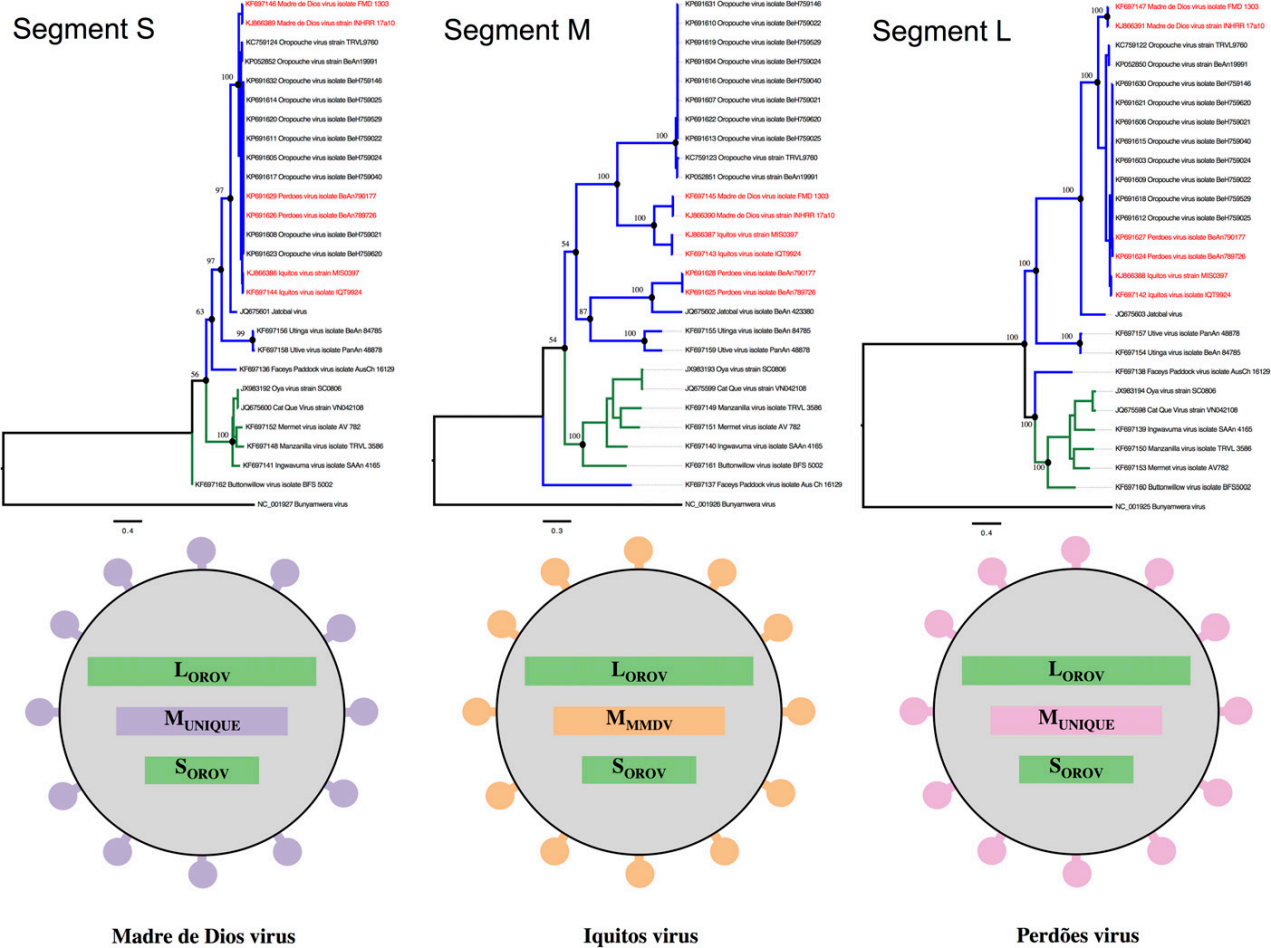
Maximum likelihood phylogenetic analysis of subclade A: Simbu serogroup showing evidences of different tree topologies suggestive of genetic reassortment between Oropouche virus (OROV). In the above panel, the branches are highlighted according to the species recognized by the ICTV, ORO species in blue and Manzanila species in green, and taxon names in red indicates the reassortment events. The below panel illustrates the genetic pattern for the reassortment events involving Iquitos virus (IQTV), Madre de Dios virus (MDDV), and Perdões virus (PERDV).

Genomic reassortment is considered to be one of the most important mechanisms for the generation of viral biodiversity in orthobunyaviruses. This phenomenon can occur when two genetically related viruses infect the same susceptible cell at the same time, and the progeny virus can be formed containing a varied mixture of genomic L, M, and S segments from the two parental viruses. This phenomenon, which is also common with segmented viruses from other families, can have significant implications due to the possibility of the emergence of a virus with increased pathogenicity. The exchange of genomic RNA segments involving OROV have been reported in isolates obtained from Peru, Venezuela, and Brazil. The first reassortment event was reported in Peru when the Iquitos virus (IQTV) was isolated in 1999 from a febrile patient.^42^ Further genome characterization of Simbu group viruses identified in 2007, also in Peru, the presence of another OROV reassortant associated to human disease, namely Madre de Dios virus (MDDV).^41^ Also, the MDDV was reported in a monkey (*Cebus olivaceus Schomburgk*) in a forest near Atapirire, a small rural village located in Anzoategui State, Venezuela.^43^ In 2015, Tilston-Lunel and others (2015) described the presence of Perdões virus (PERDV), a new OROV reassortant species isolated from the viscera of a nonhuman primate (*Callithrix* sp.) found dead in Minas Gerais State, Brazil.^22^ All reassortant species showed both SRNA and LRNA related to OROV, whereas the MRNA of IQTV probably is from MDDV, whereas the MRNAs of MDDV and PERDV remain unknown yet and are probably unique to its species (Figure 6

). It is interesting to notice that in reassortment events, usually the S and L segments are those that are exchanged between species.^16^ It is known that the nucleocapsid protein coded by the S segment and the L polymerase function as a pair to replicate the viral genome. Therefore, one segment restricts the molecular evolution of the other, and is thought to be inherited as a pair. On the other hand, the M segment, which codes for the viral glycoproteins, could be more subjected to mutations due to the higher selective pressure on its coding region, since these proteins are the major host range determinants. The hypothesis that a different rate of mutation in the M segment compared with both L and S has also been suggested by Tilston-Lunel and others,^25^ which could also explain the molecular differences observed in the M segment of the reassortant viruses.

## Clinical Aspects

Infections in humans caused by OROV are characterized as an acute febrile illness, usually accompanied by headache, myalgia, arthralgia, anorexia, dizziness, chills, and photophobia (Figure 3). Some patients present a rash that resembles rubella and nausea, vomiting, diarrhea, conjunctive congestion, epigastric pain and retro–orbital pain, and other systemic manifestations have also been described.^16^ A few days after the initial febrile episode, a recurrence of symptoms is commonly observed, but usually with less intensity, representing approximately 60% of cases. Some patients may display a clinical presentation of meningitis.^44^ Convalescence is complete without apparent sequel, even in severe cases. There is no fatality record demonstrably related to Oropouche fever.^3^,^44^ However, identification of OROV SRNA genomic segment in cerebrospinal fluid in patients from Amazonas State, northern Brazil, suggests that severe disease committing the central nervous system (CNS) is occurring during outbreaks of this virus in Brazil.^45^

## Pathogenesis

Since fatalities have not been recorded, little is known about the pathogenesis of natural OROV infections. Humans present systemic symptoms, with viremia detected in the initial 2–4 days of the onset of the first symptoms. In some patients, the virus has been recovered from the cerebrospinal fluid, but the route of invasion of the CNS is unknown.^46^

Studies using experimental animal models have helped to elucidate some aspects of the pathogenesis of OROV. In 1978, Araujo and others demonstrated that viruses could be detected in liver lesions with significant necrosis of hepatocytes and considerable hypertrophy of Kupffer cells 6 hours after OROV was inoculated intracerebrally into 3-week-old hamsters, suggesting a hematogenous transmission of the virus from the brain to the liver.^39^

A more detailed infection model in hamsters using viruses inoculated subcutaneously demonstrated the occurrence of systemic infection, with high viral load in the plasma, that can reach a peak of 10^6.0^ TCID_50_/mL at the 3rd day after infection.^39^,^47^ In this model, both brain and liver tissues demonstrated histological lesions, with the detection of viruses in high titers, and positive immunohistochemistry showing the presence of viral antigens in neurons.^47^,^48^

The pathogenesis of OROV was also studied in murine models. BALB/c neonate mice inoculated subcutaneously presented clinical signs at the 5th day after inoculation. The animals presented a high concentration of replicating virus in the brain, with inflammation of the meninges and apoptosis of neurons, apparently without encephalitis.^47^ These data confirmed the neurotropism of this virus, as observed in the hamster model. The access to the CNS was revealed by immunohistochemistry, which showed that OROV infection advances from the posterior parts of the brain toward the forebrain. OROV reaches the neural routes during the early phases of infection, reaching the spinal cord and ascending to the brain through brainstem regions, with little inflammation. During the later periods of the infective cycle the virus crosses the blood–brain barrier, spreading into the brain parenchyma, with more severe manifestations of encephalitis.^47^

Studies based on subcutaneous inoculations of OROV in wild type and mutated immunocompromised C57BL/6 mice revealed that induction of type I IFN pathway through mitochondrial antiviral-signaling protein, interferon regulatory transcription factor 3 and 7, and interferon-α/β receptor is essential to control OROV infection, and this likely occurs dominantly in nonmyeloid cells.^49^ In addition, the IRF-5 is a key component of the immune response against orthobunyaviruses and has a role in modulating the antiviral response in peripheral tissues, while contributing to inhibit the neuroinvasion process.^50^

## Laboratorial Diagnosis

Diagnosis of OROV infection is basically made using classic and molecular virology techniques: 1) virus isolation attempt in newborn mice and cell culture (Vero cells); 2) serologic assays, such as HI, NT, CF tests, and in-house-enzyme linked immunosorbent assay for total immunoglobulin, IgM and IgG detection, respectively, in convalescent sera; 3) reverse transcription polymerase chain reaction (RT-PCR) and real-time RT-PCR for genome detection in acute samples (sera, blood, and viscera of infected animals).^23^,^51^,^52^ In the latter case, the molecular methods were designed for specific detection of SRNA genome fragments. However, due to the existence of OROV reassortant events (e.g., MDDV, IQTV, and PERDV), new approaches based on the MRNA segment of OROV, which is unique to this virus, is needed to detect specifically infections caused by this important human pathogen.

In 2001, Saeed and others engineered the first nucleocapsid recombinant protein antigen using a plasmidial *Escherichia coli* bacterial system. This antigen was tested in IgM enzyme-linked immunosorbent assay (ELISA) assay in clinical samples from patients infected by the OROV in Brazil and Peru.^51^ Unfortunately, there are no available commercial diagnostic or rapid tests based on immunoassays (e.g., ELISA, immunochromatography).

## Future Perspectives

OROV is one of the most important orthobunyaviruses associated with human diseases in tropical America, with more than 30 major outbreaks and half million reported cases since its first isolation in 1955. In this review, we discussed the major breakthroughs achieved in OROV research. However, there are many more questions that need to be addressed to fully understand the epidemiology and pathogenesis of this virus. The reverse genetics system developed for OROV^25^ could be exploited for this purpose, which could be used to better understand the role of the nonstructural proteins during viral replication in mosquito and mammalian cells. This reverse genetics system could also be used to better understand the reassortment events in the family *Bunyaviridae* and its effects. Since the M segment is the only viral genomic segment that is different between MMDV, IQTV, and PERDV reassortants, the system could be used to generate recombinant viruses and study the differences they present in terms of pathogenesis, virulence outcome, and host range, which could elucidate important implications on the understanding of the evolution of orthobunyaviruses in South America. The virus-like particles assay developed for this virus^21^,^25^ could also be used as a tool to generate a potential recombinant vaccine as well as a safe BSL-3 free ELISA diagnosis, which could facilitate the study of the prevalence of OROV in South America.

## Figures and Tables

**Table 1:** Distribution and classification of members of the Simbu serogroup according to the species, virus, geographic distribution, and year of isolation

Species	Virus	Distribution	Isolated from (year)
Akabane	Akabane virus	Africa, Asia, Australia	Cattle (1974)
Akabane	Sabo virus	Africa	Goat (1966)
Akabane	Tinaroo virus	Australia	Midges (1978)
Akabane	Yaba-7 virus	Africa	Mosquitoes (1963)
Manzanilla	Buttonwillow virus	North America	Rabbits (1962)
Manzanilla	Ingwavuma virus	Africa, Asia	Birds (1959)
Manzanilla	Inini virus	South America	Birds (1973)
Manzanilla	Manzanilla virus	South America	Monkey (1954)
Manzanilla	Mermet virus	North America	Monkey (1964)
Oropouche	Facey's Paddock virus	Australia	Mosquitoes (1974)
Oropouche	Jatobal virus	South America	Coati (1985)
Oropouche	Oropouche virus	South America	Human (1955)
Oropouche	Utinga virus	South America	Sloth (1965)
Oropouche	Utive virus	South America	Sloth (1975)
Sathuperi	Douglas virus	Australia	Cattle (1978)
Sathuperi	Sathuperi virus	Africa, Asia	Mosquitoes (1957)
Sathuperi	Schmallenberg virus	Europe	Cattle (2011)
Shamonda	Peaton virus	Australia	Midges (1976)
Shamonda	Sango virus	Africa	Cattle (1965)
Shamonda	Shamonda virus	Africa	Cattle (1965)
Shuni	Aino virus	Asia, Australia	Mosquitoes (1964)
Shuni	Kaikalur virus	Asia	Mosquitoes (1971)
Shuni	Shuni virus	Africa	Cattle (1966)
Simbu	Simbu virus	Africa	Mosquitoes (1955)
Thimiri	Thimiri virus	Africa, Asia	Birds (1963)
